# Peripheral inflammatory cytokines are associated with the microstructural characteristics of the corpus callosum and prefrontal cortex as detected by magnetic resonance T1/T2 mapping in the CUMS rat model

**DOI:** 10.1016/j.heliyon.2024.e40428

**Published:** 2024-11-20

**Authors:** Li Wang, Fengying Yuan, Qiaoli Yuan, Guidong Dai, Xiaofei Lu, Li Zhou, Yurong Zheng, Yunzhu Wu, Maohua Wang, Guangxiang Chen

**Affiliations:** a Department of Radiology, The Affiliated Hospital of Southwest Medical University, Luzhou, 646000, Sichuan, China; b Department of Anesthesiology, The Affiliated Hospital of Southwest Medical University, Luzhou, 646000, Sichuan, China; c SIEMENS Healthineers Ltd., China

**Keywords:** Chronic unpredictable mild stress, Depression, Magnetic resonance imaging, Inflammatory cytokine, Kynurenine

## Abstract

**Background:**

Several clinical neuroimaging studies have reported structural and functional brain abnormalities associated with peripheral inflammatory cytokines or kynurenine pathway metabolites in patients with depression. However, it is not clear whether the abnormal findings are changes that are intrinsic to depression or whether they are caused by confounding factors such as age, illness duration, or medication.

**Methods:**

To exclude confounding factors, we used chronic unpredictable mild stress (CUMS) rat model and magnetic resonance T1/T2 mapping to investigate the microstructural characteristics of the prefrontal cortex (PFC), hippocampus and corpus callosum and further explored the association between peripheral blood depression-related indicators and microstructural characteristics.

**Results:**

The results revealed that the T2 relaxation time of the corpus callosum was significantly decreased in the CUMS model compared to the control group. Additionally, positive correlations were found between the levels of inflammatory cytokines (interleukin (IL)-1 and IL-6) and the T1 relaxation time of the corpus callosum and between the level of IL-6 and the T1 relaxation time of the PFC.

**Conclusion:**

Our study demonstrates that the microstructural abnormality of the corpus callosum is an intrinsic change that accompanies depression and also provides robust evidence that the microstructural characteristics of the corpus callosum and PFC associated with inflammatory cytokines in peripheral blood play an important role in the fundamental pathophysiological mechanism of depression.

## Graphical abstract

**Figure:**
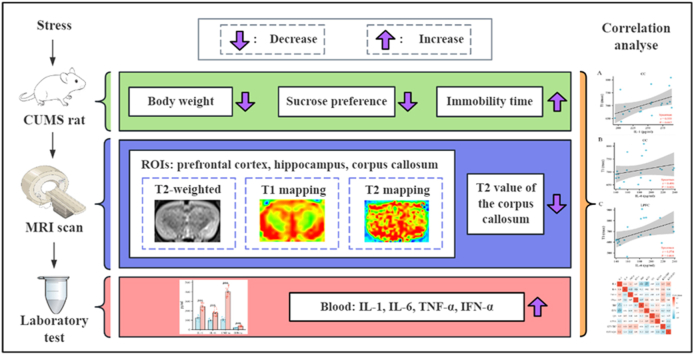

## 1 Introduction

Major depressive disorder (MDD) is a major cause of disability and is characterized by depressed mood and anhedonia, along with significant cognitive dysfunction [1,2]. Accumulating evidence from recent studies suggests that inflammatory cytokines are closely associated with the pathophysiology of depression [[3], [4], [5]]. Inflammatory cytokines, which are produced primarily by macrophages, are small signalling proteins that are upregulated during inflammation and are critical for initiating and promoting the inflammatory response in depression. Several studies have shown that the activation of the kynurenine (KYN) pathway in patients with depression is secondary to increased levels of the inflammatory cytokines interleukin (IL)-6, interferon (IFN)-α and tumor necrosis factor (TNF)-α [[6], [7], [8]]. Patients with depression have abnormal blood levels of KYN pathway metabolites, such as tryptophan (TRP), KYN, kynurenic acid (KYNA) and quinolinic acid (QA) [9,10]. As a neuroprotective factor, KYNA can antagonize the toxic effects of QA, but abnormal accumulation of KYNA may lead to glutamatergic dysfunction and cognitive dysfunction [11].

Magnetic resonance imaging (MRI) has been widely used to study alterations in the brains of patients with mental illness because of its noninvasiveness and high spatial and temporal resolution [12,13]. Several clinical neuroimaging studies have reported structural and functional brain abnormalities (e.g., in the prefrontal cortex (PFC), hippocampus, amygdala, and corpus callosum) in patients with depression. Moreover, these abnormalities have been linked to inflammatory cytokines and KYN pathway metabolism. For example, the thickness of the PFC was significantly decreased in the MDD group and inversely correlated with the serum IL-6 level [14]. In addition, the fractional anisotropy (FA) values of the genu of the corpus callosum in MDD patients are significantly reduced and negatively correlated with IL-1β levels [15]. One review reported that chronic peripheral inflammation reduces adult hippocampal neurogenesis [16]. Furthermore, increased inflammatory cytokines can predict reduced functional connectivity between the right amygdala and left ventromedial PFC [17]. In addition, the KYNA/QA ratio is positively correlated with hippocampal and amygdalar volumes in MDD patients [18,19]. Pizzagalli et al. reported that an imbalance in the KYN pathway could reduce the volume of the hippocampus and prefrontal lobe [20]. A recent study revealed that lower TRP levels are associated with lower FA in the corpus callosum in bipolar disorder patients but not in patients with unipolar depression [21]. These findings suggest that abnormalities in the PFC, hippocampus and corpus callosum play important roles in the pathophysiological mechanism of depression and that cerebral abnormalities may be associated with an imbalance in peripheral inflammatory cytokines and KYN pathway metabolites.

However, because factors such as sex, age, disease course, and treatment (such as medication regimens) of patients included in clinical studies are difficult to standardize and because some patients may not have incomplete information available, it is not clear whether the abnormal findings result from changes that are intrinsic to depression or whether they result from confounding factors. In animal models, these confounding factors can be controlled more readily than they are in clinical patients; thus, animals are often used to investigate the physiopathological mechanisms of diseases. The classical depression model most commonly used in research is the chronic unpredictable mild stress (CUMS) model, which was first applied to rats in 1981 by Katz and colleagues [22]. The CUMS model can induce anhedonia and is therefore regarded as a realistic model of depression.

Only a few MRI studies have reported abnormalities in brain structure and function in a rat model of depression on the basis of the CUMS exposure paradigm. Regional brain abnormalities are located mainly in stress-sensitive brain regions, namely, the hippocampus, PFC, corpus callosum, and caudate–putamen [[23], [24], [25], [26]]. Interestingly, structural MRI revealed inconsistent results regarding the hippocampal volume of the CUMS model [23,25,27]. Luo and colleagues reported that the hippocampal volume of CUMS rats was decreased and was negatively associated with learning and memory changes [27]. However, two other studies reported no significant differences in hippocampal volume between the CUMS and control groups [23,25]. A diffusion tensor imaging study revealed signs of demyelination in the left hippocampus, left caudate nucleus, globus pallidus, corpus callosum, bilateral frontal lobe and hypothalamus [26]. One magnetic resonance spectroscopy study revealed significant decreases in glutamate and glutamine levels in both the hippocampus and the PFC, whereas elevated levels of myo-inositol and taurine were observed only in the hippocampus of CUMS-induced animals [24]. MRI T1 and T2 mapping can be used to analyse the microstructural characteristics of biological tissues quantitatively through changes in longitudinal and transverse relaxation times, from which the T1 and T2 values can be quantified; these techniques have good stability and reliability. To date, the application of this imaging technology has expanded from the cardiovascular system [28] to the liver [29], kidney [30], brain [31] and other tissues and organs. To the best of our knowledge, no study has used this technique to investigate changes in brain microstructure and the relationships between these changes and depression-related peripheral blood variables in a CUMS model.

In this study, we aimed to investigate the microstructural characteristics revealed by magnetic resonance relaxation times via T1 and T2 mapping techniques in several crucial brain regions (the PFC, hippocampus and corpus callosum) and the alterations in peripheral inflammatory factors and KYN pathway metabolites in a CUMS model. In addition, we explored whether depression-related indicators in peripheral blood influence the microstructural characteristics of these brain regions in this model.

## 2 Materials and methods

### 2.1 Experimental subjects

A total of 70 specific-pathogen-free male Sprague–Dawley (SD) rats (SYXK (Sichuan) 2018–065), aged 6–8 weeks and weighing 200–240 g each, were purchased from the Animal Experimental Center of Southwest Medical University and included in this study. The rats in the control group were housed in cages, whereas the rats in the model group were housed in a single cage in the standard experimental environment (except for the cage feeding required by the CUMS model). The temperature was 22 ± 1 °C, the relative humidity was 40–60 %, the light cycle was maintained (except when disrupted for the purpose of the CUMS model), and adequate food and water were provided (except when food and water were needed for the CUMS model). The experiments were conducted in accordance with the recommendations of the National Institutes of Health Guidelines for Animal Studies and were approved by the Ethics Committee of Southwest Medical University.

### 2.2 Experimental materials

The materials for the experiment were as follows: sucrose (KESH), a bucket for forced swims; a flashlight; an ultrasonic cleaner; a clamp; cotton; syringes; saline; isoflurane (RWD); an anaesthetic gas evaporator (PENLON); a Siemens PRISMA 3.0 T magnetic resonance scanner; an Agilent HPLC-1100; and a TSKgel OSD-100 V column.

### 2.3 Experimental methods

**CUMS rat model construction and weight measurement.** Seventy specific-pathogen-free-grade male SD rats aged 6–8 weeks were fed adaptively for 2 weeks, and 13 rats were excluded for abnormal baseline behavior in the SPT and FST. At a ratio of 1:3, the rats were randomly divided into a control group (n = 15) and a CUMS model group (n = 42). The CUMS model rats were exposed to different types of unpredictable mild stressors for 8 weeks, whereas the control group was not. The stressors were applied in a random sequence and included stroboscopic light, cold swimming (5 min, 4 °C), hot swimming (5 min, 45 °C), physical restraint (4 h), noise (4 h), food and water deprivation (24 h), tail pinch (5 min), and cage shaking combined with wet bedding (24 h). The body weights of the rats were measured weekly.

**Sucrose preference test (SPT).** The SPT is used to assess anhedonia, a decrease in the ability to experience pleasure; this abnormality is one of the most common clinical manifestations of depression. At 72 h before the baseline SPT, all the rats were subjected to sucrose adaptation training: two bottles of 1 % sucrose were placed in the cage for the first 24 h, one bottle of 1 % sucrose was replaced with water for the second 24 h, and the rats were fasted and deprived of water for the third 24 h. After acclimation training, all the rats were subjected to the baseline SPT, for which a 1 % sucrose solution and water were prepared and used. The initial weights of the filled sucrose and water bottles were measured and recorded. In the experiment, the positions of sucrose and water were changed to eliminate spatial bias. After 24 h, the bottles were reweighed to calculate the amount of each liquid consumed, and the sucrose preference rate was calculated as follows: sucrose preference rate (%) = consumption of sucrose solution/(consumption of sucrose solution + consumption of plain water). The SPT was performed before, during and after model generation.

**Forced swimming test (FST).** The FST assesses despair-like behavior by measuring the latency of an animal to become immobile when it is placed in water. A transparent cylindrical Plexiglas swimming bucket (bottom diameter 20 cm × height 50 cm) was partially filled with water at an appropriate temperature (24 ± 1 °C); the depth of the water was approximately 30 cm. The rats were lowered directly into the water from a short distance, and the activity of the rats in the next 5 min was recorded by a camera. After the completion of the test, the rats were removed, the water in the swimming bucket was discarded, any remaining rat excreta were removed from the bucket, and fresh water was added in preparation for the next rat. At the end of the experiment, DIGBEHVE software was used to analyse the swimming behavior of the rats, and their immobility time was used to evaluate their degree of despair. The FST was performed before, during and after model generation.

**Fig. 1:**
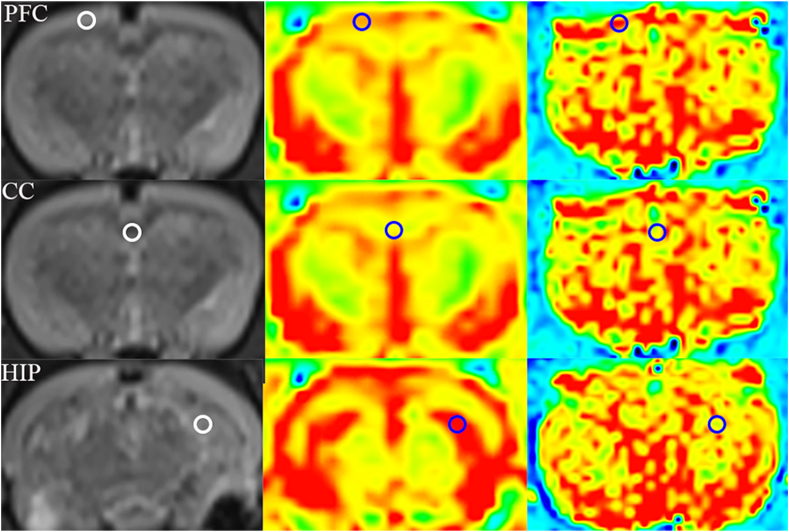
Regions of interest in T2-weighted, T1 mapping and T2 mapping sequence images. CC = corpus callosum; PFC = prefrontal cortex; HIP = hippocampus.

**MRI scanning.** MRI scans were performed via a MAGNETOM Prisma 3.0 T MRI scanner (Siemens Healthcare, Erlangen, Germany) with a wrist coil. The 2D T2-weighted turbo spin‒echo images were acquired as follows: repetition time (TR) = 3660 ms, echo time (TE) = 91 ms, flip angle = 120°, field of view (FOV) read = 60 mm, FOV phase = 100 %, and slice thickness = 2 mm. The scanning parameters for T1 mapping were as follows: TR = 6 ms, TE = 2 ms, flip angle = 3°, FOV read = 136 mm, FOV phase = 100 %, and slice thickness = 2 mm. The T2 mapping scanning parameters were as follows: TR = 1000 ms, TE = 11.8 ms, flip angle = 180°, FOV read = 160 mm, FOV phase = 100 %, and slice thickness = 2 mm. Five regions of interest (ROIs) of the bilateral PFC, hippocampus and corpus callosum were drawn on the T2-weighted structural images via a Siemens postprocessing workstation. The values of T1 and T2 were measured by aligning the T2-weighted structural image ROIs to the T1 and T2 maps. Fig. 1 shows the T2-weighted structural images, T1 mapping and T2 mapping from left to right.

**Laboratory tests.** Immediately after the MRI scans, blood samples were taken from both groups of rats by opening the neck under isoflurane gas anaesthesia and drawing blood from the internal jugular vein. Serum samples were retrieved after the blood was centrifuged at 3000 r/min, and the levels of the inflammatory cytokines IL-1, IL-6, TNF-α and IFN-α in the blood were detected via enzyme-linked immunosorbent assay (ELISA). The levels of TRP, KYN, KYNA and QA were detected via high-performance liquid chromatography (HPLC).

### 2.4 Statistical methods

SPSS 17 was used for statistical analysis. For comparisons of numerical variables between two groups, a normality test was performed first. If the data from each group were normally distributed and the variance was equal between the two groups, the mean ± standard error of the mean was used for statistical description, and a *t*-test was used for comparisons between groups. Otherwise, the median (with the interquartile range) was used for statistical description, and the Mann‒Whitney *U* test was used for comparisons between groups. The Spearman correlation coefficient was used for correlation analysis. *P* < 0.05 was considered statistically significant.

## 3 Results

### 3.1 Body weight

**Fig. 2:**
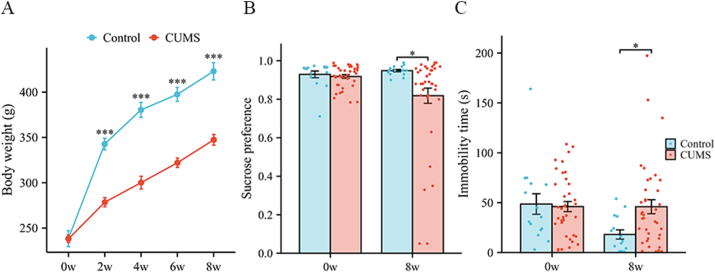
Changes in body weight and behavioral test indices. A Changes in body weight (g). B Changes in the sucrose preference rate. C Changes in immobility time (s). Error bars indicate SEM. N = 15 in the control group, N = 38 in the CUMS group. ∗P < 0.05, ∗∗∗P < 0.001, ns represents "not significant". CUMS = chronic unpredictable mild stress.

At the baseline stage (0 weeks), there was no significant difference in body weight between the depression model rats and the blank control rats. From the second to the eighth week of CUMS, the body weights of the control group rats increased more significantly than those of the depression model rats did (*P* < 0.001), as shown in Fig. 2A and Table 1.

**Table 1:** Changes in the weights of the rats in the two groups before and after CUMS intervention (g).

	0 w	2 w	4 w	6 w	8 w
Control (15)	238.33 ± 8.86	342.71 ± 6.36	380.39 ± 8.28	397.49 ± 7.71	422.99 ± 9.57
CUMS (38)	238.17 ± 4.19	278.52 ± 5.08	300.18 ± 7.01	322.08 ± 5.17	347.41 ± 5.90
Z	−0.36	−4.90	−4.80	−5.07	−4.47
*P*	0.722	0.000	0.000	0.000	0.000

The data are expressed as the mean ± SEM. CUMS = chronic unpredictable mild stress.

### 3.2 Behavioral tests

**SPT.** At the baseline level before model generation, there was no statistically significant difference in the percentage of sucrose preference between the depression model rats and the blank control rats. Compared with that of the control rats, the sucrose preference ratio of the depression group rats significantly decreased after CUMS was applied (*P* = 0.016), as shown in Fig. 2B.

**FST.** Before stress stimulation, there was no statistically significant difference in the FST immobility time between the depression model rats and the blank control rats, but after stress stimulation, the FST immobility time of the depression group rats was significantly greater than that of the control rats (*P* < 0.001), as shown in Fig. 2C.

### 3.3 Magnetic resonance relaxation times in T1 and T2 mapping

**Fig. 3:**
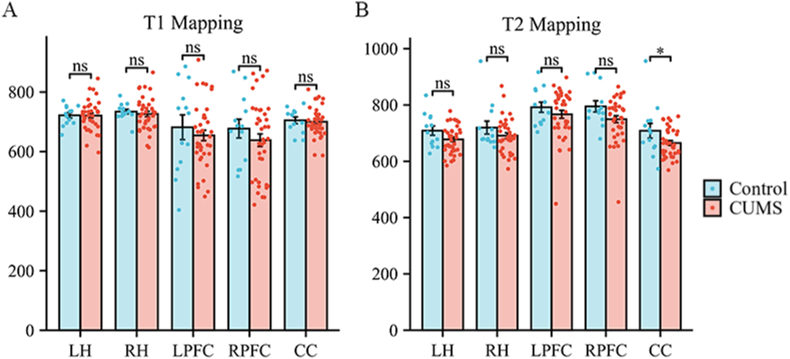
Changes in the T1 and T2 relaxation times. A Changes in T1 relaxation times. B Changes in T2 relaxation times. Error bars indicate SEM. N = 13 in the control group, N = 38 in the CUMS group. ∗P < 0.05, ns represents "not significant". CC = corpus callosum; CUMS = chronic unpredictable mild stress; LH = left hippocampus; LPFC = left prefrontal cortex; RH = right hippocampus; RPFC = right prefrontal cortex.

At the end of the 8-week model generation process, the two groups of rats underwent MRI scanning. The values of T1 and T2 in the bilateral hippocampus, PFC and corpus callosum were measured. The results revealed that the T2 value of the corpus callosum of the rats in the depression group was significantly lower than that of the control group (*P* = 0.043), whereas the T1 value of the corpus callosum was not significantly different between the two groups (*P* > 0.05). There was no significant difference in the T1 or T2 values in the bilateral hippocampus or PFC between the two groups (*P* > 0.05), as shown in Fig. 3.

### 3.4 Peripheral inflammatory factor and KYN pathway metabolites

**Fig. 4:**
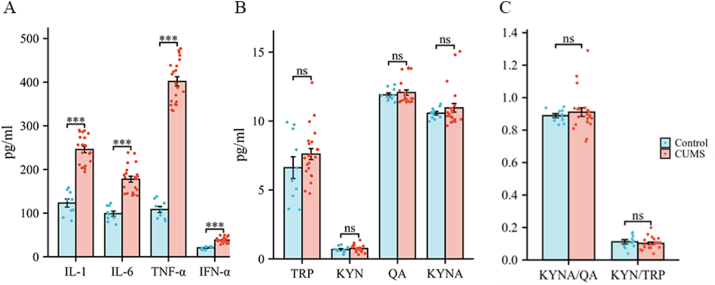
Changes in peripheral inflammatory cytokines and KYN pathway metabolites. A Changes in peripheral inflammatory cytokines. B Changes in peripheral KYN metabolites. C Changes in the KYNA/QA and KYN/TRP ratios of KYN metabolites. Error bars indicate SEM. Peripheral inflammatory cytokines: N = 9 in the control group, N = 21 in the CUMS group; KYN pathway metabolites: N = 10 in the control group, N = 22 in the CUMS group. ∗∗∗P < 0.001, ns represents "not significant". IFN = interferon; IL = interleukin; KYN = kynurenine; KYNA = kynurenic aid; QA = quinolinic acid; TNF = tumor necrosis factor; TRP = tryptophan.

**IL-1, IL-6, TNF-α, and IFN-α.** The CUMS group presented significantly increased blood levels of the following peripheral inflammatory factors: IL-1 (*P* < 0.001, CUMS = 245.67 ± 7.25, Control = 122.97 ± 9.29), IL-6 (*P* < 0.001, CUMS = 177.67 ± 6.84, Control = 98.71 ± 5.82), TNF-α (*P* < 0.001, CUMS = 408.60 ± 10.75, Control = 108.20 ± 7.01), and IFN-α (*P* < 0.001, CUMS = 38.21 ± 1.42, Control = 20.46 ± 1.14). The detailed data are described in Fig. 4A and Table 2.

**Table 2:** Changes in blood inflammatory cytokines (pg/ml).

	IL-1	IL-6	TNF-α	IFN-α
Control (9)	122.97 ± 9.29	98.71 ± 5.82	108.20 ± 7.01	20.46 ± 1.14
CUMS (21)	245.67 ± 7.25	177.67 ± 6.84	408.60 ± 10.75	38.21 ± 1.42
*P*	0.000	0.000	0.000	0.000

The data are expressed as the mean ± SEM. CUMS = chronic unpredictable mild stress; IFN = interferon; IL = interleukin; TNF = tumor necrosis factor.

**KYN Pathway Metabolites.** No significant differences in the blood levels of the KYN pathway metabolites TRP (*P* > 0.05, CUMS = 7.60 ± 0.40, Control = 6.62 ± 0.79), KYN (*P* > 0.05, CUMS = 0.75 ± 0.05, Control = 0.67 ± 0.07), QA (*P* > 0.05, CUMS = 12.07 ± 0.19, Control = 11.90 ± 0.14), or KYNA (*P* > 0.05, CUMS = 10.96 ± 0.31, Control = 10.60 ± 0.13) were detected between the CUMS and control groups. See Fig. 4B. Similarly, there were no significant group differences in the ratios of KYN/TRP (*P* > 0.05, CUMS = 0.10 ± 0.04, Control = 0.11 ± 0.04) or KYNA/QA (*P* > 0.05, CUMS = 0.91 ± 0.03, Control = 0.89 ± 0.01), as shown in Fig. 4C.

### 3.5 Correlation analyses

**Fig. 5:**
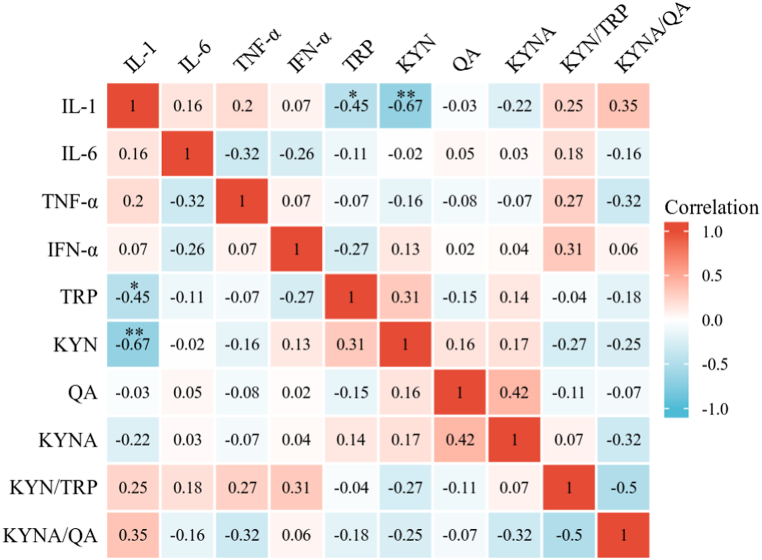
Correlation analysis between peripheral inflammatory cytokines and KYN pathway metabolites. N = 21 in the CUMS group. ∗P < 0.05, ∗∗P < 0.01. IFN = interferon; IL = interleukin; KYN = kynurenine; KYNA = kynurenic acid; QA = quinolinic acid; TNF = tumor necrosis factor; TRP = tryptophan.

**Correlation analyses of inflammatory factors and KYN pathway metabolites.** Correlation analyses revealed that IL-1 was significantly negatively correlated with KYN (R = −0.665, *P* = 0.001) and TRP (R = −0.451, *P* = 0.040) in the blood of CUMS model rats, as shown in Fig. 5. There were no correlations between other inflammatory factors and metabolites (*P* > 0.05).

**Correlation analyses between behavioral and blood** indices. Correlation analyses revealed that the behavioral indices (sucrose preference rate and FST immobility time) were not significantly correlated with blood inflammatory factors or metabolites in CUMS model rats (*P* > 0.05).

**Correlation analyses between behavioral indices and T1/T2 values.** Correlation analyses revealed that there were no significant correlations between behavioural indices (sucrose preference rate, FST immobility time) and the T1/T2 values of the bilateral PFC, hippocampus or corpus callosum in CUMS model rats (*P* > 0.05).

Correlation analyses between blood indices and T1/T2 values.

**Fig. 6:**
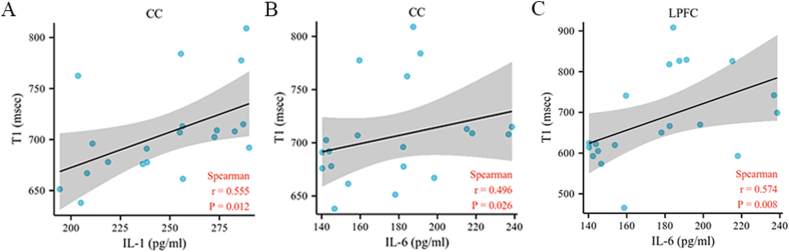
Correlation analysis between peripheral inflammatory cytokines and T1 relaxation times. Correlation analysis between the IL-1 and T1 relaxation times of the CC. B Correlation analysis between the IL-6 and T1 relaxation times of the CC. C Correlation analysis between the IL-6 and T1 relaxation times of the LPFC. N = 20 in the CUMS group. CC = corpus callosum; IFN = interferon; IL = interleukin; LPFC = left prefrontal cortex; TNF = tumor necrosis factor.

Correlation analyses revealed that the levels of IL-1 and IL-6 in the blood of CUMS model rats were positively correlated with the T1 value of the corpus callosum (R = 0.555, *P* = 0.012; R = 0.496, *P* = 0.026) and that the level of IL-6 was significantly positively correlated with the T1 value of the left PFC (R = 0.574, *P* = 0.008), as shown in Fig. 6. Other inflammatory factors and metabolites were not significantly correlated with the T1/T2 values (*P* > 0.05).

## 4 Discussion

In this study, the levels of the inflammatory cytokines IL-1, IL-6, TNF-α, and IFN-α were significantly increased in the peripheral blood of CUMS model rats, whereas the levels of KYN pathway metabolites (TRP, KYN, KYNA, and QA) and the KYN/TRP and KYNA/QUIN ratios were not significantly different. Our study also revealed that IL-1 was significantly negatively correlated with KYN and TRP. Moreover, the present study demonstrated that the T2 value of the corpus callosum decreases in CUMS model rats. Positive correlations were detected between the levels of inflammatory cytokines (IL-1 and IL-6) and the T1 value of the corpus callosum and between the level of IL-6 and the T1 value of the left PFC.

The behavioral test results revealed that the CUMS model rats had a significantly reduced preference for sucrose and increased immobility time in the FST, which confirmed that the model group had common symptoms of depression, such as anhedonia and behavior despair. The body weights of the rats in the model group decreased significantly in the first week of the CUMS procedure, which indicated that the CUMS group of rats consumed fewer unpredictable stressors than did the control group at the beginning of the first week. Overall, the changes in body weight, sucrose preference in the SPT, and immobility time in the FST confirmed the success of the CUMS procedure.

As an innate immune response to tissue injury, neuroinflammation is believed to be closely associated with the pathophysiology of MDD [[32], [33], [34]]. In recent years, a series of studies have noted that biological indicators in peripheral blood samples, mainly IL-1, IL-6, TNF-α, IFN-α, C-reactive protein, neurotrophic factor and KYN pathway metabolites, can be analysed as criteria for the diagnosis of depression [6,35,36]. One of the widely accepted hypotheses is that chronic stress stimulation of the sympathetic nervous system leads to dysregulation of the hypothalamic‒pituitary‒adrenal (HPA) axis, which increases the production of inflammatory cytokines in peripheral blood and activates indoleamine 2,3-dioxygenase (IDO) activity, promoting a transition from serotonin (5-hydroxytryptamine, or 5-HT) synthesis to KYN pathway metabolite synthesis through the action of inflammatory cytokines [37,38]. We observed that CUMS rats had higher levels of peripheral inflammatory cytokines (IL-1, IL-6, TNF-α, and IFN-α) than did the control group, which is consistent with this theory. Moreover, we found that there were no significant differences in KYN pathway metabolites in the peripheral blood of rats in the model group compared with those in the control group. This finding is consistent with a meta-analysis showing that the levels of IL-6 and IL-8 in cerebrospinal fluid were significantly increased in patients with MDD, whereas no significant difference was found in the KYN pathway metabolites [39]. However, the increase in peripheral IL-1 in the model group was associated with decreases in KYN and TRP. These results indicate that activated inflammatory cytokines may play a pivotal role in the development of depression by downregulating KYN and TRP. In general, these findings may suggest that researchers should focus their future studies not on any single marker but on the combination of inflammatory factors and KYN pathway metabolites as biomarkers for depression.

Magnetic resonance T1 and T2 mapping techniques can reflect cell density, water content, polypeptides, proteins and other macromolecules. In terms of magnetic resonance brain imaging studies in depression models, researchers have mostly used magnetic resonance tensor and spectral techniques and found abnormalities in brain regions such as the hippocampus, corpus callosum and PFC [26,40]. Our study using T1 and T2 mapping techniques revealed that the T2 values of the corpus callosum were significantly lower in the depression model rats than in the control rats. One possible explanation is the abnormal accumulation of iron in the corpus callosum of CUMS model rats. Iron, as a paramagnetic substance, generates a spin magnetic moment that increases the strength of the local magnetic field, leading to a nonuniform increase in the magnetic field and accelerated proton dephasing, thus shortening the transverse relaxation time [[41], [42], [43]]. Moreover, clinical studies have shown that brain iron deposition is one of the main neuropathological hallmarks of MDD, which also provides a basis for this hypothesis [44,45].

The corpus callosum is the largest compact white matter fibre bundle in the brain and consists of myelinated axons that connect the left and right hemispheres, facilitating interhemispheric communication. It plays a key role in subserving the transfer of sensory, motor, and cognitive information between the two hemispheres, supporting coordinated brain function [46,47]. Specifically, the fibres connecting the PFC pass through the anterior part of the corpus callosum to transmit motor information between the two hemispheres and coordinate motor functions. Additionally, the anterior section of the corpus callosum, with its finer associative fibres, is essential for complex cognitive processing, as it connects nonhomologous regions of the cerebral cortex. In contrast, the PFC, another brain region revealed by our study to be associated with inflammatory processes, is critical for higher-order cognitive processes, including emotion regulation, memory management, and executive functions [20,48]. Despite their distinct roles, the corpus callosum and the PFC are functionally interconnected. The corpus callosum enables communication between the PFCs of both hemispheres, which is essential for tasks that require the integration of information from both sides of the brain [49]. For example, the corpus callosum facilitates the coordination of sensory input and motor output across hemispheres, supporting seamless execution of tasks. Notably, owing to the functional differences between the left and right frontal lobes, the communication changes between the PFC induced by the destruction of the corpus callosum may cause greater cognitive impairment, highlighting the importance of this connection for integrated brain function and increased cognitive processes [50,51].

Previous studies have reported microstructural changes in the corpus callosum in patients with depression [52,53]. Our findings obtained via magnetic resonance T1/T2 mapping are consistent with previous results regarding microstructural alterations in the corpus callosum, as revealed by clinical and animal model studies. Furthermore, our study revealed that the levels of IL-1 and IL-6 were positively correlated with the T1 value of the corpus callosum and that the level of IL-6 was positively correlated with the T1 value of the left PFC. These findings suggest that inflammation may affect the microstructural properties and functional connectivity of the corpus callosum and PFC, which are crucial for maintaining integrated and coordinated brain function, and that their disruption could contribute to the development of depression. Diffusion tensor imaging studies also revealed that FA values of the corpus callosum in MDD patients were inversely associated with IL-1β [15], IL-6 [54], and TNF-α levels [55]. Previous studies have shown that dysfunctions in serotonin receptors and the serotonin system in the PFC are critical pathophysiological bases of depression. Positron emission tomography studies have revealed significant decreases in blood flow and glucose metabolism in the PFC of MDD patients [56,57]. Veeraiah P et al. reported that the glutamatergic and gamma-aminobutyric acid activity levels of the PFC were dysregulated in a rat model of depression [58]. Taken together, these findings suggest that stress-induced inflammatory processes might influence the microstructural characteristics of the corpus callosum and PFC, which play key roles in the occurrence and development of depression.

One of the limitations of this study is that only male SD rats were studied, meaning that there may be sex bias if an attempt is made to extrapolate the results to the clinic. Second, the circadian rhythm of the rats may have some influence on the behavioral results. Finally, longitudinal MRI studies were not performed, as repeated scans might have had adverse effects on the rats.

## 5 Conclusion

By using CUMS model rats to exclude confounding factors, the present study demonstrated that microstructural abnormalities in the corpus callosum, as revealed by magnetic resonance T1/T2 mapping, are intrinsically involved in the pathophysiologic mechanism of depression. Moreover, the microstructural characteristics of the corpus callosum and PFC are related to inflammatory cytokines in the peripheral blood of CUMS model rats. In conclusion, the microstructural characteristics of the corpus callosum and PFC, which are associated with inflammatory cytokines in peripheral blood, play important roles in the fundamental pathophysiological mechanism of depression.

## CRediT authorship contribution statement

**Li Wang:** Writing – original draft, Investigation, Formal analysis, Data curation. **Fengying Yuan:** Methodology, Formal analysis, Data curation. **Qiaoli Yuan:** Software, Methodology, Investigation. **Guidong Dai:** Software, Methodology, Data curation. **Xiaofei Lu:** Software, Investigation, Data curation. **Li Zhou:** Visualization. **Yurong Zheng:** Visualization. **Yunzhu Wu:** Software, Methodology. **Maohua Wang:** Supervision, Project administration, Conceptualization. **Guangxiang Chen:** Writing – review & editing, Project administration, Funding acquisition, Conceptualization.

## Data availability statement

The data will be made on request.

## Ethical statement

The Ethics Committee of Southwest Medical University, No. 20211123-053.

## Funding statement

This study was supported by the Sichuan Science & Technology Department of China (Grant No. 2022YFS0616), the 10.13039/501100004912Sichuan University & Luzhou Collaborative Foundation (Grant No. 2017CDLZ-G27, 2018CDLZ-11), the Luzhou Science & Technology Department (Grant No. 2022-SYF-60), the Luzhou Science & Technology Department & 10.13039/501100014895Southwest Medical University Collaborative Foundation (Grant No. 2018LZXNYD-ZK02), the Project of 10.13039/501100020207Health Commission of Sichuan Province (Grant No. 20PJ128) and the Project for Doctors of 10.13039/501100015375Affiliated Hospital of Southwest Medical University (Grant No. 2018-17129).

## Declaration of competing interest

The authors declare that they have no conflicts of interest.
